# Beyond Clinical Examination: Utilizing MRI Surveillance to Detect Recurrence of Soft Tissue Sarcomas and Differentiate from Posttherapeutic Changes

**DOI:** 10.3390/biomedicines12081640

**Published:** 2024-07-23

**Authors:** Felix R. M. Koenig, Alfred H. Kielburg, Snehansh Roy Chaudhary, Christian Wassipaul, Akash Ganguly, Raoul Varga, Martin L. Watzenboeck, Iris-Melanie Noebauer-Huhmann

**Affiliations:** 1 Department of Biomedical Imaging and Image-Guided Therapy, Medical University of Vienna, Waehringer Guertel 18-20, 1090 Vienna, Austriairis.noebauer@meduniwien.ac.at (I.-M.N.-H.); 2 Oxford University Hospitals NHS Foundation Trust, University of Oxford, Oxford OX2 0JB, UK; 3 Warrington & Halton Hospitals NHS Foundation Trust, Warrington WA5 1QG, UK; akash.ganguly@nhs.net

**Keywords:** sarcoma, locoregional neoplasm recurrence, soft tissue neoplasms, magnetic resonance imaging, surveillance

## Abstract

Background: Early detection of soft tissue sarcoma (STS) recurrence is essential; however, the role and timeline of Magnetic resonance imaging (MRI) surveillance are still under debate. The aim of this study was to determine whether local recurrence (LR) could be identified via clinical examination alone and to assess the MRI morphology of primary STS and LR. Methods: This retrospective study included all patients with STS recurrence after surveillance for at least five years from the tumor database of the Medical University of Vienna from 2000 until December 2023. The characteristics of primary STS and LR and the time interval to recurrence and clinical detectability were assessed. The MRIs of LR and posttherapeutic changes (PTC) were compared with the initial MRIs. Results: A total of 57 patients (60% male; mean age 58.5 ± 18.0 years) with STS and histologically confirmed LR were included. The mean time interval to LR was 2.3 ± 1.8 years (range 108 to 3037 days). The clinically detectable recurrences were significantly larger than the inapparent ones (71.9 cm^3^ vs. 7.0 cm^3^; *p* < 0.01). The MRI morphology of all LRs (26/26) closely resembled the initial STS. For comparison, nine patients were included with clinically suspected LRs, which were histologically proven to be PTC. None of these resembled the primary STS. Conclusion: Based on clinical symptoms alone, especially small and early recurrences can be missed, which supports the importance of MRI surveillance.

## 1. Introduction

Soft tissue sarcomas (STSs) are a rare and heterogenous group of tumor entities with a Europe-wide incidence of 5.6/100,000/year [1]. According to Brennan et al., the most common entities are liposarcoma (20%), followed by leiomyosarcoma (14%), and undifferentiated pleomorphic cell sarcoma [2]. The 5-year and 10-year survival rates for STS are 73% and 68%, respectively [3].

Various imaging modalities are employed in the management of local STS surveillance. Ultrasound is often used as the first modality by experienced sonographers, especially for local recurrence (LR) in the extremities. However, potential issues include limited comparability of serial exams and the fact that some areas, especially those in deep locations and those involving joint regions, are not easily accessible. Positron emission tomography (PET) or computed tomography (CT) scans provide valuable additional metabolic information and are particularly useful for staging and response assessment. However, smaller recurrences without significant metabolic activity may be missed due to suboptimal spatial resolution and sensitivity of the PET component and lower soft tissue contrast compared to Magnetic resonance imaging (MRI). MRI has become the gold standard for STS evaluation due to its superior soft tissue contrast, multiplanar capabilities, and ability to provide detailed information on tumor characteristics and extent without ionizing radiation [4,5].

In cases of recurrence, the 5-year survival rate is significantly reduced to 53% [6]. Overall, STS LR rates of 17% and 20% have been described at 5 and 10 years. In contrast, LR of extremity STS is relatively uncommon after adequate surgical resection and subsequent radiotherapy, with recurrence rates of 9% at 5 years and 12% at 10 years post-surgery [7]. However, the risk of LR increases with positive or unclear resection margins, deep location in the trunk or head and neck area, age over 64 years, tumor size > 10 cm, high tumor grade, and history of prior LR [8]. Certain tumor types, such as undifferentiated pleomorphic sarcoma, myxofibrosarcoma, neurogenic or epithelioid sarcoma, and dedifferentiated liposarcoma in the retroperitoneum also exhibit a propensity for more frequent recurrences [3]. The spread of the primary tumor beyond its original compartment also fosters LR [9].

Currently, there are no established evidence-based guidelines for sarcoma surveillance using imaging. It is presumed that most LR manifests as pain or palpable resistance. Thus, the precise role of follow-up imaging is ambiguous [10,11]. Nevertheless, the detection of non-palpable or clinically inconspicuous recurrence hinges on follow-up imaging [12]. High-risk patients typically experience recurrences within 2–3 years, whereas for low-risk patients, they are less frequent and generally occur later [8].

Recently, the European Society of Musculoskeletal Radiology (ESSR) guidelines for imaging STS were updated to establish recommendations for standardized imaging of the primary tumor [13]. Current guidelines recommend follow-up MRI to detect LR in STS patients depending on tumor grade [14]. Notably, the early identification of recurrence is of paramount importance in predicting the outcomes of individuals with STS. However, the utilization and optimal timing of MRI surveillance remain subjects of ongoing discussion.

This study aimed to evaluate the MRI characteristics of STS recurrence, draw comparisons with the primary tumor, ascertain the feasibility of detection solely through clinical examination, and gauge the duration between the initial diagnosis of the primary STS and recurrence.

## 2. Materials and Methods

This retrospective study was approved by the local institutional review board, and the need for informed consent was waived (Ethics Committee 2159/2017).

### 2.1. Study Population

Patients listed in the Orthopedic Tumor Database at the Medical University of Vienna with primary STS and corresponding surveillance MRI were included if they met the following inclusion criteria: (1) histology of a primary STS; (2) surgery for resection of primary STS (with or without neoadjuvant or adjuvant radiotherapy or chemotherapy); (3) availability of MRI examinations; (4) histological confirmation of LR or posttherapeutic changes (PTC); (5) surveillance of at least five years after the primary diagnosis; (6) MRI scans were included with a field strength of at least 1.5 Tesla. The minimum required imaging protocol consisted of the following sequences: an axial T2w sequence, a fluid-sensitive fat-saturated sequence, and T1-weighted sequences both before and after contrast administration. The exclusion criteria were (1) a lack of documented follow-up; (2) an absence of sufficient quality of follow-up MRI; (3) a lack of current histological examination. Patient data were pseudonymized.

**Figure 1:**
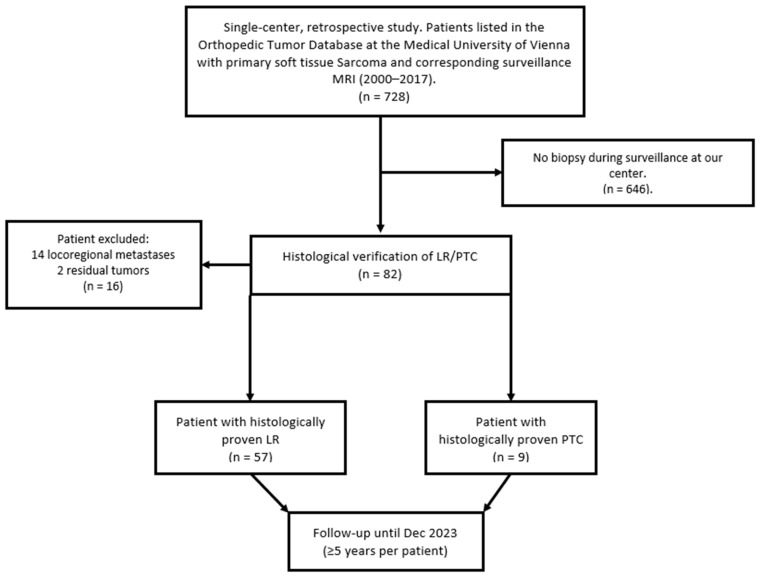
This flowchart depicts the patient selection process for this retrospective study on STS, detailing the inclusion of patients with histologically verified LR and PTC; LR, Local Recurrence; PTC, Posttherapeutic Changes.

This database consisted of 728 patients with primary STS diagnosed between January 2000 and December 2017. During the observation period, 82 patients underwent biopsies for suspected LR or regional metastasis. Of these, 57 patients showed a histopathologically verified LR. In 14 patients, the histologic result was locoregional metastasis, two patients had a residual tumor, and in nine patients, histology revealed benign PTC. Patients were followed up until December 2023, resulting in a surveillance period of at least five years for each patient. A flowchart is provided in Figure 1.

### 2.2. Assessment

The following histological and clinical parameters were assessed: histology of the primary tumor, initial tumor grade, localization, presence of metastasis at the initial diagnosis, and metastasis in the course of the disease as well as the history of treatment.

The volume of LR was examined by measuring the LR in three axes and calculating its volume, assuming an ellipsoidal shape, using the formula V = length × width × height × π/6.

STS LRs were classified as deep if located beneath the deep muscle fascia and superficial if situated above it (epifascial). Standardized MRI surveillance and assessment of the clinical history regarding pain or new swelling in the surgical area were carried out. In our center, we use a standardized MRI protocol for post-treatment follow-up, as described in the ESSR soft tissue tumor imaging guidelines [14]. However, this study included patients whose primary MRIs were performed elsewhere, leading to protocol heterogeneity. Primary MRI refers to baseline imaging before initiation of therapy. Clinical examination, regional MRI, and chest CT were performed in a standardized manner, as described in the ESSR guidelines [14], every three to four months (grading dependent) during the first three years after treatment, every six months after three to five years, and once a year between five and 10 years after treatment. If LR was suspected outside of this schedule, these patients were examined clinically, and a timely follow-up MRI was scheduled. Histology (intraoperative or a biopsy) was performed on suspected masses. Clinical examination involved physical palpation of the original STS site and assessment of patient-reported symptoms, including pressure sensation, paresthesia, and pain. LR was considered clinically apparent if a palpable mass was detected or if the patient reported any related symptoms; otherwise, it was deemed clinically inapparent. To retrospectively compare clinical detectability in LR and PTC, clinical symptoms and palpability, if clinically determined, were analyzed. PTC were defined as imaging changes observed following surgery and/or radiotherapy, encompassing both expected healing processes (e.g., swelling, bruising) and complications (e.g., infection, hematoma, and cystic changes). All postoperative findings included in our study were histologically confirmed to be free of residual STS. Patients were followed for a minimum of five years, during which no local recurrence was observed.

In histologically verified LR and PTC, the following MRI parameters were assessed: signal intensity of the lesions in T1- and T2- weighted sequences, homogeneity, margins, lobulation, post-contrast enhancement and contrast enhancement homogeneity, necrosis, and size. T1 and T2 signal intensity, homogeneity, and post-contrast enhancement patterns were compared, if possible, with the initial MRI appearance of the primary STS. If all these features were similar, the tumor was categorized as having a similar appearance compared to the primary. If at least one of the features was different, it was categorized as having a different appearance.

### 2.3. Statistical Analysis

The statistical analysis was performed using SPSS version 24.0.1 for Windows (IBM,, Chicago, IL , USA). Continuous variables are expressed as mean ± standard deviation or as median [interquartile range], as appropriate. Categorical variables are expressed as absolute numbers and their percentages. The figures were generated using GraphPad Prism version 10 (GraphPad Software, La Jolla, CA, USA, https://www.graphpad.com/, accessed on 15 December 2023) and SPSS.

Survival outcomes were assessed using the Kaplan–Meier method. The non-parametric Shapiro–Wilk test was used to determine the distribution of the data (*p* < 0.05). Therefore, a Mann–Whitney U-test and Wilcoxon signed ranks test without multiplicity were applied to prevent an increase in type two errors. *p*-values equal to or below 0.05 were considered statistically significant.

## 3. Results

### 3.1. Patient Population

The final cohort consisted of 57 patients with confirmed LR after STS. The mean age at diagnosis of the primary STS was 58.5 ± 18.0 years (range 12 to 92 years), and 59.6% (34/57) of the patients were male.

Nine patients were also suspected for LR but had histologically proven benign PTC. Their mean age at diagnosis of primary STS was 58 ± 17.7 years (range 35 to 82 years), with 44.4% (4/9) being male. None of these patients developed LR or locoregional metastasis during surveillance up until December 2023, with a mean follow up time of 8.88 ± 2.15 years (range 5.65 to 12.30 years).

The patients’ characteristics of LR and PTC at the time of the primary STS diagnosis are summarized in Table 1.

Among the verified LR, the most frequent primary histology was myxofibrosarcoma, accounting for 29.8% of the primaries, followed by liposarcomas with 19.3%, undifferentiated pleomorphic sarcomas (14%), leiomyosarcomas (8.8%), and synovial cell sarcomas (5.3%). Additionally, there were four cases of NOS (not otherwise specified) sarcoma, three cases of fibrosarcoma, and two cases each of malignant mesenchymoma and spindle cell sarcoma. One case of each of the following entities was histologically verified: fibromyxoid sarcoma and angiosarcoma.

Initial analysis of the confirmed LRs showed that 49.1% of the primary tumors were poorly differentiated (high grade) (G3) vs. 21.1% that were intermediate (G2), and only 8.8% were well differentiated (low grade) (G1) [15]. In 21.1% of the cases, initial grading could not be determined due to factors such as external resection or prior chemotherapy, while 14% (8/57) of the patients had presented with metastases at the time of the primary tumor diagnosis, and 43.9% (25/57) of the patients developed metastases during the course of their disease.

All patients included in this study had undergone surgical resection. Neoadjuvant therapy was administered to 68.4% (39/57) of the LR patients and 55.6% (5/9) of the PTC patients. Adjuvant therapy was received by 40.4% (23/57) of the LR patients and 33.3% (3/9) of the PTC patients. In the LR group, 68.4% (39/57) of the patients had undergone radiation therapy, and 40.4% (18/57) had received chemotherapy. In the PTC group, 8 (89%) patients had undergone radiation therapy, and 4 (44%) patients had received chemotherapy. The initial surgical resections had been classified as “marginal” (0.1–2 mm) in 38 patients (66.7%). Margins with >2 mm distance of healthy tissue to the tumor had been achieved in 8 patients (14.0%). Two surgical margins (3.5%) had been classified as “intralesional.” In 9 patients who had undergone surgery elsewhere, the surgical margins had not been documented (15.8%).

### 3.2. LR and Clinical Detectability

Clinical information was available for 41 patients with later histologically proven LRs (71.9%). Clinical examination data were not available for 16 patients of the LR group. LR was clinically apparent as a palpable mass in the area of the original STS in 70.7% (29/41) of the patients. In these patients, 86% (*n* = 25) reported a feeling of pressure, 10% (*n* = 3) additional paresthesia, and 28% additional (*n* = 8) pain. In 12 patients (29.3%), no palpable mass was found on clinical examination. None of these patients reported clinical symptoms (such as a feeling of pressure, paresthesia, or pain), and the LR could only be identified by MRI.

LR was clinically inapparent in 27% (7/26) of the patients who had initially received radiotherapy and in 33.3% (5/15) of the patients who had not received radiotherapy.

A total of 29/37 (66.7%) LRs in the lower extremity were clinically apparent, as well as 7/8 (87.5%) in the upper extremity and 6/9 (66.7%) in the trunk, without a significant association between location and clinical detectability (*p* = 0.509).

**Figure 2:**
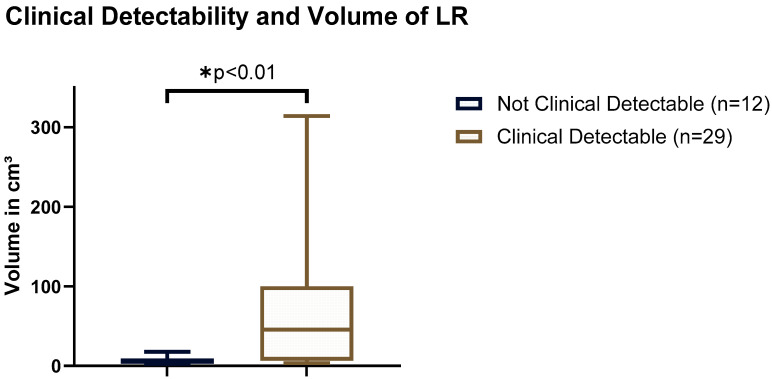
Clinical detectability and volume of LR. Significant difference in mean volume of clinically detectable (71.9 cm^3^) and not clinically detectable LR (7.0 cm^3^). LR, Local Recurrence. * indicates that the result is significant with a p-value of less than 0.01.3.3. Period between Primary STS and LR.

Clinically detectable LRs were located superficially in 6/29 (20.7%), while 23/29 (79.3%) of the LRs were deep. Of note, 6/7 (86%) of the superficially located LRs were clinically detectable, while this was the case in only 23/34 (68%) of the deep LRs. No significant difference was seen between the depth of the LR and clinical detectability (*p* = 0.339). See Table 2. Clinically symptomatic LRs showed a significantly larger mean volume of 71.9 ± 15.7 cm^3^ compared to 7.0 ± 1.5 cm^3^ in the asymptomatic patients (*p* < 0.01, Mann–Whitney U-test); Figure 2.

In five patients with PTC, clinical information was available. All those patients presented with clinical symptoms and a palpable resistance (5/5).

**Figure 3:**
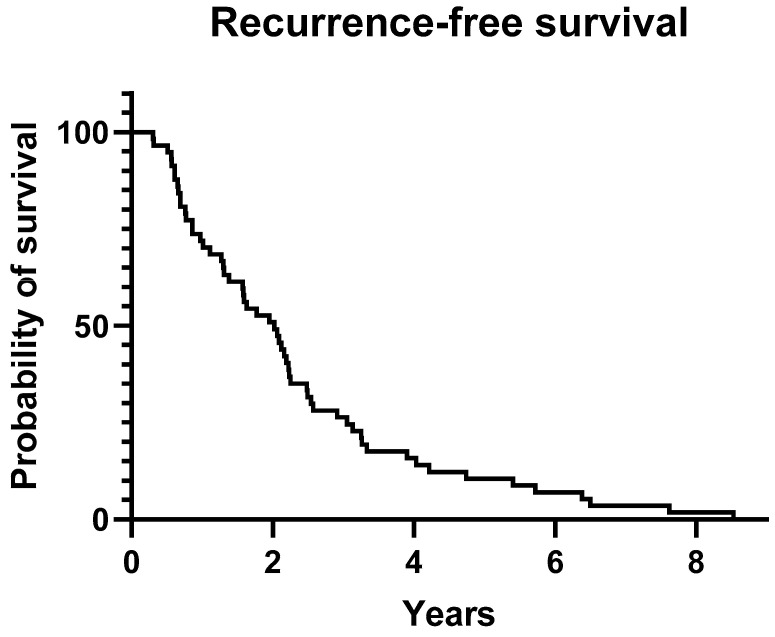
Recurrence-free survival: 50% of LR happened within 2.02 years; the last LR took place after 8.5 years.

The mean time between the diagnosis of initial STS and the histologically verified LR was 832.3 ± 666.7 days (2.3 ± 1.8 years, *n* = 57, range 108–3037 days), with 50% of LRs occurring after 2.02 years, and 74% within 3 years. For visualization, a Kaplan–Meier curve is provided in Figure 3.

Among the 45 patients for whom information about the grading of the primary STS was available, there was no significant difference between the grading and recurrence-free survival (G1 (*n* = 5/45); G2 (*n* = 12/45); G3 (*n* = 28/45); *p* = 0.173).

### 3.3. MRI Features of LR Compared to the Primary MRI

**Figure 4:**
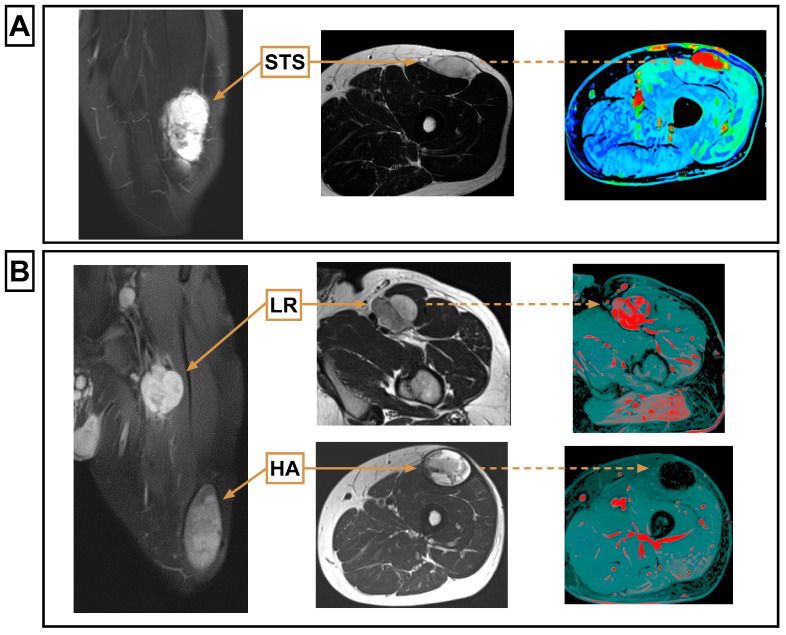
Histologically verified STS showing the same T1-weighted (w) signal intensity, T2-weighted signal intensity, homogeneity, and contrast enhancement as the histologically verified LR that occurred during the course of the disease. Using these four features allows MR tomographic differentiation of LR and PTC. A newly occurring hematoma can be distinguished from an LR on the basis of the different contrast agent uptake and on the basis of the different homogeneity. (**A**) STS, from left to right: HASTE coronal, T2w axial, KM flooding of the primary tumor; (**B**) LR and HA, from left to right: T1 fat suppression post contrast coronal, upper row: LR: T2w axial, subtraction of the LR; lower row: T2w axial, subtraction of the HA. HA, hematoma; LR, local recurrence; STS, soft tissue sarcoma.

All LRs (26/26) that could be compared with the primary STS showed a matching MR morphology regarding the T1-weighted signal intensity, T2-weighted signal intensity, homogeneity, and contrast enhancement. Detailed results are provided in Table 3. Imaging examples are provided in Figure 4.

MRI features of all PTC (*n* = 9) differed from those of the primary STSs in at least one of the above-mentioned features. Their MRI features were as follows: On T1-weighted sequences, 6 cases were hypointense, and 3 were hyperintense. On T2-weighted sequences, 8 cases were hyperintense, and 1 was hypointense. Most cases (6/9) were inhomogeneous and showed contrast enhancement (8/9) (Table 3).

## 4. Discussion

In this retrospective study, the clinical detectability of STS LRs was examined, and the MR features were compared with the primary tumor.

Finding an individualized follow-up strategy for patients after STS treatment continues to be a clinical challenge, and evidence-based data are scarce. Blaye and others have reported that in one third of patients, metastases or an LR were detected due to clinical symptoms [16]. It is even under debate whether imaging should be performed at fixed intervals or only in symptomatic cases [10,11].

In this study, 29% of the patients had clinically asymptomatic LRs. Our findings highlight the potential benefits of personalized follow-up schedules for STS patients, even in the absence of LR symptoms. These results are consistent with other recent studies, Hovgaard et al. showed that local imaging in the first two years after STS surgery detects LR and lung metastases better than clinical examination and X-ray [17]. Furthermore, England et al. demonstrated that one third of LR was only detected by imaging [18].

The clinical detectability of STS LR in different locations is widely discussed. Blaye et al. do not recommend routine MRI surveillance to identify LR of sarcomas affecting the limbs and trunk wall, but recommend using imaging resources for the assessment of primary tumor sites for which evaluation by history and physical examination alone is inadequate [16]. In our population, 33% of the lower extremity and 12% of the upper extremity LRs were clinically undetectable but revealed by MR imaging. Irrespective of the high cost/benefit ratio for these regular MRI examinations [19], our data show that the high number of asymptomatic LRs underlines the clinical benefit of routine MR examination for follow-up, including the extremities. Other studies support our hypothesis. Routine local imaging follow-up already showed an improved survival rate in high-risk patients [20,21].

Clinical detectability also depends on the depth of STS LR, since deep LR is often clinically inapparent [22]. However, in this study, we did not find a significant relationship between the depth of the LR and its clinical detectability. This may be due to the small number of cases in our study. Nevertheless, the symptomatic LRs were significantly larger compared to the asymptomatic LRs. The studies by Rothermundt et al. and Whooley et al. recommend clinical surveillance only, but these studies did not report the size of the LRs [23,24]. Our results clearly emphasize the benefit of routine surveillance MRI at fixed intervals in patients with STS for detecting especially smaller, thus early LR, which are still asymptomatic.

All LRs in our study showed the same morphologic MRI parameters as the primary STS. Therefore, a lesion resembling the primary tumor during follow-up examinations should raise urgent suspicion of LR. This emphasizes the importance of comparing surveillance MRI with preoperative imaging and supports the recommendation to conduct STS treatment at specialized centers [14,25]. Dynamic contrast-enhanced (DCE) MRI was not included in the analysis because it was only available in a minority of patients, although Hirschmann et al. reported that it improved detection of LR [26].

Among the PTC that had been clinically suspicious for LR, none showed similarity to the primary tumor in MRI. As such, the differentiation between LR and PTC remains a formidable clinical hurdle, which can be eased by MRI follow-up.

The time between surgery and LR averaged around 2.3 years (27.7 months), and 74% of the LRs occurred within three years, while the longest interval to LR was 8.5 years. Only a few studies exist regarding the mean recurrence-free interval after STS. Lewis et al. reported a mean recurrence-free period of 18 months [27]. However, since their study is from 1997, (neo)-adjuvant treatment options have changed fundamentally, which may explain the difference. Our data support the recommendation by the ESSR to perform imaging checkups for up to 10 years following the initial treatment in high-risk patients [14]. Especially in the first three years, narrow intervals are suggested (three to four months for high-grade STS and four to six months for low-grade STS) [14].

### Limitations and Strengths

This study contributes new knowledge to the field of LR after STS by comparing clinical detectability, MRI imaging, and histology. Our approach has several major strengths. First, for a rare entity such as STS, the patient cohort was relatively large. Second, as a specialized tumor center, we used standardized and long-term patient follow-up. Finally, these results support post-treatment follow-up MRI and comparison with protoperative MRI for accurate surveillance diagnosis. Taken together, these elements enhance our understanding of the role of MRI in the diagnosis and monitoring of LR, potentially improving patient care.

This study has several limitations. Firstly, the study was conducted in a single center, and local practice or patient demographics may have confounded the results. Second, the retrospective nature of this study and the incomplete clinical data limited the cohort size and did not allow individual evaluation of the different STS entities. Thirdly, comparison between the MRI of the primary STS and its LR was only possible in about 45% of the patients, and only nine patients with PTC could be included. Protocols were heterogeneous because primary MRI was often not performed at our center. To obtain a comparable cohort of patients, we included patients with absolutely necessary MRI requirements as described in the Methods section. DCE was also only available in a minority of patients and could not be included in our analysis. An ideal MRI protocol, which we recommend and use in our center, is described in the ESSR 2023 soft tissue tumor imaging guidelines [13].

To conduct a more comprehensive analysis, a broader cohort of patients would be required. In conclusion, almost a third of LRs were clinically undetectable but identified by routine MRI surveillance; all LRs displayed the same morphologic MRI characteristics as the primary STS. Therefore, routine post-treatment follow-up MRI and comparison with preoperative MRI should be mandatory to allow for accurate surveillance diagnosis and to address LR early.

## Figures and Tables

**Table 1:** Patient Characteristics at primary STS diagnosis (*n* = 66).

Characteristic		Local Recurrence (*n* = 57)	Posttherapeutic Changes (*n* = 9)	Total (*n* = 66)
Age at Diagnosis (Mean ± SD)		58.5 ± 18.0 years	58.5 ± 17.70 years	58.5 ± 18.0 years
Sex	Male:	34 (40%)	4 (44%)	38 (58%)
	Female:	23 (60%)	5 (56%)	28 (42%)
Location of Primary Tumor	Lower Extremity:	37 (65%)	6 (67%)	43 (65%)
	Upper Extremity:	9 (16%)	2 (22%)	11 (17%)
	Trunk:	11 (19%)	1 (11%)	12 (18%)
Primary Histology	Liposarcoma:	11 (19%)	2 (22%)	13 (20%)
	Myxofibrosarcoma:	17 (30%)	2 (22%)	19 (29%)
	Undifferentiated Pleomorphic Sarcoma:	8 (12%)	0	8 (12%)
	Synovial Cell Sarcoma:	3 (5%)	2 (22%)	5 (8%)
	Leiomyosarcoma:	5 (9%)	1 (11%)	6 (9%)
	Other:	13 (23%)	2 (23%)	15 (23%)
Primary Grading	No Grading Available:	12 (21%)	6 (67%)	18 (27%)
	G1:	5 (9%)	0	5 (8%)
	G2:	12 (21%)	1 (11%)	13 (20%)
	G3:	28 (49%)	2 (22%)	30 (46%)
Metastasis at Diagnosis	Yes:	8 (14%)	1 (11%)	9 (14%)
	No:	49 (86%)	8 (89%)	57 (86%)
Neoadjuvant Therapy	Yes:	39 (68%)	5 (56%)	44 (59%)
	No:	18 (32%)	4 (44%)	22 (41%)
Adjuvant Therapy	Yes:	23 (40%)	3 (33%)	26 (35%)
	No:	34 (60%)	6 (66%)	60 (65%)

This table summarizes the baseline characteristics of all patients included in the study.

**Table 2:** Clinically detectable and not clinically detectable LR of STS (*n* = 41). Clinical data were not available for 16 patients.

	Clinically Detectable LR (*n* = 29)	Not Clinically Detectable LR (*n* = 12)	Total (*n* = 41)
Volume of LR (cm^3^, mean ± SD)			44.59 ± 63.7
	71.9 ± 15.7	7.0 ± 1.5	
	
Location of LR (*n*, %)	Superficial: 6 (20.7) Deep: 23 (79.3)	Superficial: 1 (8.3) Deep: 11 (91.7)	Superficial: 9 (15.8) Deep: 48 (84.2)
Time intervall between primary diagnosis and LR (years, mean ± standard deviation)	2.3 ± 1.8 (range 108 to 3037 days)		

Characteristics of LR among patients, categorized into clinically detectable and not clinically detectable. Clinical data were not available for 16 patients. The time interval between STS and LR was defined as the time between initial diagnosis of STS and histologic confirmation of LR.

**Table 3:** MRI features of LR and PTC compared to baseline MRI at primary STS diagnosis.

		Local Recurrence (*n* = 26)		Posttherapeutic Changes (*n* = 9)	
		Count	%	Count	%
T1w intensity	Hypointense	18	69	6	67
	Hyperintense	8	31	3	33
T2w intensity	Hypointense	1	4	1	11
	Hyperintense	25	96	8	89
Homogeneity	Inhomogeneous	15	58	6	67
	Slightly inhomogeneous	4	15	1	11
	Homogeneous	7	27	2	22
Contrast agent uptake	No contrast enhancement	0	0	1	11
	Contrast enhancement	26	1	8	89
Contrast agent uptake	No contrast enhancement	0	0	1	11
	Contrast enhancement	26	1	8	89
Overall MRI feature similarity		Similar to primary STS in all features (26/26)		Differed from primary STS in at least one feature (9/9)	

MRI features of LR and PTC, comparing their signal intensities, homogeneity, and contrast uptake with the baseline MRI at primary STS diagnosis. It highlights the similarity of LR features to the primary STS diagnosis, while PTC features differ in at least one aspect.

## Data Availability

The anonymized participant data on which this study is based will be made available upon request by email to the corresponding author by qualified researchers after publication. No imaging data exceeding the images provided in this manuscript can be made available. Proposals will be approved by the authors on the basis of scientific merit and absence of competing interests after the signing of a data access agreement and confidentiality agreement.

## Abbreviations

CT	Computed tomography
DCE	Dynamic contrast-enhanced
ESSR	European Society of Musculoskeletal Radiology
HA	Hematoma
LR	Local recurrence
MRI	Magnetic resonance imaging
NOS	Not otherwise specified
PET	Positron emission tomography
PTC	Posttherapeutic changes
STS	Soft tissue sarcoma
w	Weighted
